# Preservation of Ejaculatory Function After Postchemotherapy Retroperitoneal Lymph Node Dissection (PC-RPLND) in Patients With Testicular Cancer: Template vs. Bilateral Resection

**DOI:** 10.3389/fsurg.2018.00080

**Published:** 2019-01-17

**Authors:** Andreas Hiester, Alessandro Nini, Anna Fingerhut, Robert große Siemer, Christian Winter, Peter Albers, Achim Lusch

**Affiliations:** Department of Urology, Medical Faculty, University of Duesseldorf, Duesseldorf, Germany

**Keywords:** germ cell cancer, non-seminoma, retroperitoneal surgery, chemotherapy, bilateral and template resection

## Abstract

**Background:** Post-chemotherapy retroperitoneal lymph node dissection (PC-RPLND) plays a crucial role in treatment of metastatic non-seminomatous germ cell cancer.

**Objective:** To evaluate the functional outcome regarding the preservation of ejaculatory function comparing a bilateral vs. unilateral template resection in PC-RPLND patients. In addition, oncological safety and perioperative complications of the unilateral template resection was compared to the full bilateral one.

**Design/Setting/Participants:** Between 2003 and 2018, 504 RPLNDs have been performed in 434 patients. The database of consecutive patients was queried to identify 171 patients with PC-RPLND after 1st line chemotherapy for a non-seminoma with or without bilateral template resection. Re-Do's, late relapse, salvage patients, and thoraco-abdominal approaches were excluded. Indication for a template resection was a unilateral residual mass mainly <5 cm as published (1).

**Outcome, Measurement, and Statistical Analysis:** Descriptive statistics were used to report preoperative features, postoperative outcomes and patterns of recurrence, on the overall population and after stratification for the type of resection (bilateral vs. unilateral). Kaplan-Meier analyses were used to describe recurrence- and cancer-specific mortality-free survival rates at different time points.

**Results and Limitations:** Overall, 90 and 81 patients underwent unilateral and bilateral radical resection, respectively. Median size of residual mass was 7 cm for bilateral and 4 cm for unilateral template resection. Clinical stage II and III were present in 31 and 69% of patients, respectively. Median follow-up was 14.5 months (IQR 3.3–37.6). The 1- and 2-year recurrence-free survival rates were 91 and 91%, and 77 and 72% for patients treated with unilateral template and bilateral resection, respectively (*p* = 0.0078). Median time to recurrence was 9.5 and 9 months in template and bilateral resection group, respectively.

Adjunctive procedures were performed in 56 patients (33%) and were significantly more frequent in the bilateral resection group (43 vs. 23%, *p* = 0.006). The overall high-grade complication rate (Clavien-Dindo ≥ III) was 6, 3, and 9% in unilateral template and bilateral resection group, respectively (*p* = 0.6). The rate of preservation of antegrade ejaculation was significantly higher in the unilateral group.

**Conclusions:** Antegrade ejaculation in patients undergoing unilateral template resection with a residual mass <5 cm can be preserved at a much higher rate. Moreover, this surgical procedure is oncologically safe in terms of mid-term recurrence and CSM-free survival rates. This data undermines the growing evidence of limited PC-RPLND being justifiable in strictly unilateral residual mass <5 cm. This data has to be confirmed with a longer follow-up regarding in-field and retroperitoneal recurrences.

## Introduction

Testicular cancer is a curable disease as long as treatment is performed in specialized centers (2). In patients with advanced tumor stage a multidisciplinary treatment with chemotherapy and subsequent surgery is necessary (3, 4). The aim of adjuvant surgery after chemotherapy is to remove all vital tumor cells including teratoma. In 10–20% of all cases histopathology reveals vital cancer and in 30–40% postpubertal teratoma. The remaining patients show necrosis (3).

With a worldwide incidence of about 70,000, testicular cancer is a rare disease (5, 6). According to the current EAU and NCCN Guidelines on testicular cancer PC-RPLND is mandatory in non-seminoma patients with a residual mass larger than 1 cm (7, 8). In patients with residual mass <1 cm after chemotherapy, PC-RPLND is optional as relapse rate amounts to 6–10% and mature teratoma is seen in about 40% of cases (9–12). As both the EAU and NCCN Guidelines on testicular cancer recommend a fully bilateral resection (7, 8) several study groups have shown that unilateral template resection might achieve equal oncological results than a bilateral resection (1, 13, 14). The main reason to perform a unilateral template resection are represented by increasing the rate of antegrade ejaculation preservation and by lowering complication rate.

The aim of the present study is to compare perioperative and oncological outcomes between PC-RPLND unilateral template and bilateral resection in a retrospective analysis of a contemporary cohort of patients in a single institution. Additionally, the functional outcome regarding preservation of ejaculatory function comparing both surgical approaches (bilateral vs. unilateral) was evaluated.

## Methods

### Study Population

All patients underwent PC-RPLND after cisplatin-based chemotherapy for non-seminomatous germ-cell cancer at a single tertiary referral center. Complete data on patients' characteristics, like age at first diagnosis, histological data on orchiectomy, serum tumor markers at first diagnosis, as well as clinical stage according to the International Germ Cell Cancer Collaborative Group (IGCCCG) were recorded.

In this study, only primary PC-RPLNDs were included, secondary PC-PLNDs, salvage RPLNDs, or RPLNDs for late-relapse were excluded as well as thoraco-abdominal approaches. Prior to chemotherapy, all patients underwent a standardized staging with thoraco-abdominal CT scan, evaluation of serum tumor markers (ß-HCG, AFP). Within six weeks after termination of chemotherapy all patients underwent thoraco-abdominal CT scan and evaluation of serum tumor markers. At PC-RPLND all patients had normalized or plateauing serum tumor markers. CT scans were reviewed by a radiologist and according to size and location of primary and residual mass(es), initial histology and chemotherapy treatment associated complications bilateral or modified template approach was performed.

Perioperative outcomes were defined as percentage of preservation of antegrade ejaculation, operative time (OT), intraoperative blood loss, intraoperative complications, length of hospital stay (LoS), and postoperative complications [classified according to Clavien-Dindo classification (15)], as well as type and frequency of adjunctive surgeries. Preservation of antegrade ejaculation was recorded either by postal follow-up or during postoperative outpatient visit. The last postal follow for preservation of antegrade ejaculation was performed in 2015. Oncological outcomes were represented by type of recurrence and by recurrence- and cancer-specific mortality (CSM)—free survival rates.

### Anatomical Boundaries for PC-RPLND

The site, extent and technique performed for PC-RPLND has been discussed and described by several working groups (1, 16, 17).

**Figure 1:**
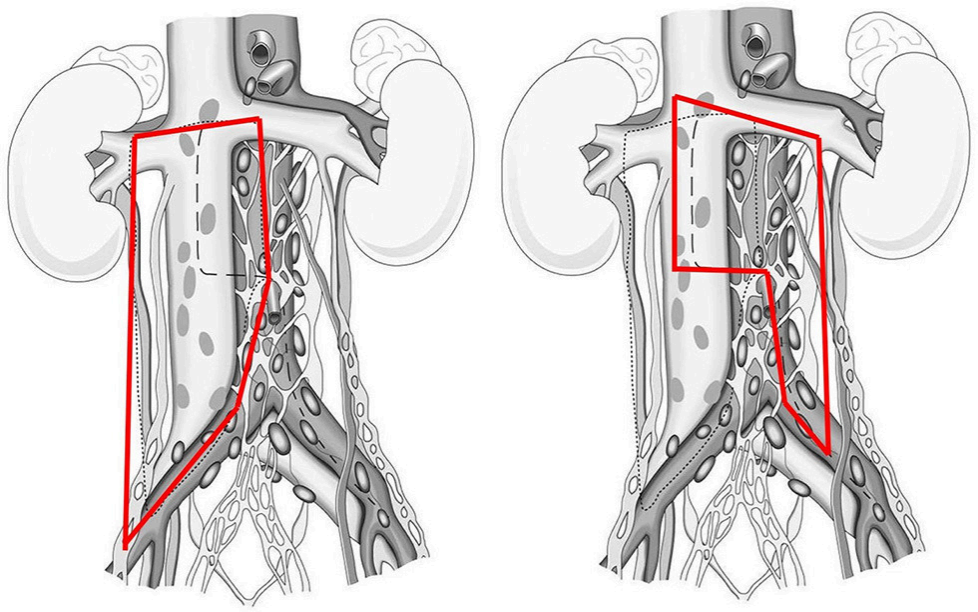
Graphical description of anatomical boundaries of right and left template PC-RPLND.

A modified template resection of the right side included the precaval, caval, paracaval, retrocaval, and inter-aortocaval regions, as well as the region lateral of the common iliac vessels. The ipsilateral ureter represented the caudal and lateral boundary of resection. In patients without retrocrural or suprahilar lymph nodes the renal vein was the cranial resection boundary (Figure 1).

On the left side, a modified template resection included the pre-aortic, retro-aortic, and para-aortic lymph nodes. The cranial boundary was the left renal artery. Medially, the superficial interaortocaval and preaortal lymph nodes up to the inferior mesenteric artery were removed with nerve-sparing of the right sympathetic nerves. Lateral and caudal boundary was represented by the ureter and the ureter crossing the vessels. In patients with radical template resection both sides were removed as described above (Figure 1). If possible, ipsilateral sympathetic nerves in all unilateral approaches (at least in the lower field of dissection, L3–L4) were preserved in order to improve the rate of antegrade ejaculation. In bilateral approaches, an attempt was sought to preserve as much of the sympathetic nerves as possible.

The decision whether to perform a unilateral template or a fully bilateral resection was taken on the base of size and location of initial and residual tumor. Patients with a residual mass of <5 cm were considered for unilateral template resection as it was demonstrated that larger masses bear a higher risk of contralateral teratoma (18–21). Patients with a residual mass >5 cm were considered for radical resection.

### Analysis

Statistical analyses, as well as reporting and interpretation of the results, consisted of two steps. Firstly, means, medians and interquartile ranges, and frequencies and proportions were reported for continuous and categorical variables, respectively, on the overall study population and after stratification for the type of resection used. Kruskal Wallis and Pearsons' Chi-square tests were used to compare medians and proportions. Secondly, Kaplan-Meier analyses were used to assess recurrence- and CSM-free survival at different time points.

All statistical tests were performed using SPSS (IBM Corp. Released 2013. IBM SPSS Statistics for Windows, Version 22.0. Armonk, NY: IBM Corp.) All tests were two-sided with a significance level set at *p* value < 0.05.

### Statement

The study was carried out following the recommendations of the University of Duesseldorf after internal approval by the University of Duesseldorf Ethical Committee. All subjects gave written informed consent in accordance with the Declaration of Helsinki.

## Results

### Patients Characteristics

Between 2003 and 2018, 504 RPLNDs for germ-cell cancer have been performed in 434 patients at one tertiary referral center. Out of these patients, 171 patients undergoing primary PC-RPLND for non-seminoma germ-cell cancer after cisplatin-based chemotherapy were identified. Baseline characteristics of the patients are shown in Table 1. The median age was 31 years (IQR 24–41). Overall, 90 and 81 patients underwent unilateral template and bilateral resection, respectively. The median size of residual mass was 7 cm (IQR 4–10) in patients undergoing bilateral resection and 4 cm in unilateral template resection (IQR 2.5–6). In the unilateral template resection group 17/90 patients (18.8%) had a residual mass of larger than 5 cm and in the bilateral resection group 17/81 patients (21%) showed residual tumor mass <5 cm in diameter.

**Table 1:** Descriptive statistics on the overall population and after stratification according to type of resection.

	**Total (*n* = 171)**	**Template (*n* = 90)**	**Bilateral (*n* = 81)**	***p*-value**
Median age, yr (IQR)	31 (24–41)	31 (24–40)	32 (24–42)	0.5
Median diameter, cm (IQR)	5 (3–8)	4 (2.5–6)	7 (4–10)	<0.001
Median AFP before PC-RPLND, μg/l (IQR)	3.3 (2.3–5.8)	3.1 (2.2–4.8)	4.4 (2.4–9.3)	0.02
Median ßHCG before PC-RPLND, IU/l (IQR)	0.1 (0.1–0.9)	0.15 (0.1–0.4)	0.1 (0.1–2.7)	0.004
**IGCCCG CLASSIFICATION**				
Good prognosis	54 (32%)	36 (40%)	18 (22%)	0.01
Intermediate prognosis	53 (31%)	29 (32%)	24 (30%)	0.7
Poor prognosis	64 (37%)	25 (28%)	39 (48%)	0.006
**UICC CLASSIFICATION AT DIAGNOSIS**				
I	14 (8%)	9 (10%)	5 (6%)	0.4
II	44 (26%)	29 (32%)	15 (18%)	0.04
III	113 (66%)	52 (58%)	61 (73%)	0.02
**UICC CLASSIFICATION BEFORE PC-RPLND**				
II	53 (31%)	36 (40%)	17 (21%)	0.007
III	118 (69%)	54 (60%)	64 (79%)	0.007

The median AFP for unilateral template and bilateral resection was 3.1 μg/l (IQR 2.2–4.8) and 4.4 μg/l (IQR 2.4–9.3) (*p* = 0.02). The median ß-HCG for unilateral template and bilateral resection was 0.15 IU/l (IQR 0.1–0.4) and 0.1 IU/l (IQR 0.1–2.7) (*p* = 0.04).

About one third of patients (32 %) were classified as “good prognosis,” 31%, as “intermediate prognosis,” and 37% as “poor prognosis” according to IGCCCG classification. A significantly higher number of patients undergoing bilateral resection were classified as “poor prognosis” compared to the unilateral template resection group (48 vs. 28%, *p* = 0.006).

Looking at the Lugano classification 53 patients (31%) presented with clinical stage II disease and 118 patients (69%) showed clinical stage III. Overall, 14 patients (8%) were classified as clinical stage I, underwent active surveillance protocol and developed a recurrence, requiring chemotherapy.

The most frequently administered chemotherapy scheme was cisplatin, etoposide, and bleomycin (PEB, 122 patients, 71%), followed by the cisplatin, etoposide, and iphosphamide scheme (PEI, 39 patients, 23%), and cisplatin and etoposide (PE, 7 patients, 4%). Three patients (2%) underwent primary high-dose (HD) chemotherapy with HD-PEI (Table 2).

**Table 2:** Descriptive statistics of the type of chemotherapy administered before surgery on the overall population and after stratification for the type of PC-RPLND.

	**Overall (*n* = 171)**	**Template (*n* = 90)**	**Bilateral (*n* = 81)**	***p*-value**
PEB	122 (71%)	66 (73%)	56 (69%)	0.5
PE	7 (4%)	6 (7%)	1 (1%)	0.1
PEI	39 (23%)	17 (19%)	22 (27%)	0.2
HD-CT (HD-PEI)	3 (2%)	1 (1%)	2 (2%)	0.9

### Perioperative Outcome, Complications, and Adjunctive Surgery

Complications and operative outcome data are shown in Table 3. Median OT was 180 min (IQR 150–210) and 240 min (IQR 180–270) for template and bilateral resection, respectively (*p* < 0.001). Median blood loss was higher for the bilateral resection group (1,400 ml, IQR 450–2500) compared to the unilateral template resection group (300 ml, IQR 100–700, *p* < 0.001). The median LoS was 9 days (IQR 8–19) with a significant shorter stay for template resection (8 vs. 9 days, template vs. bilateral, *p* = 0.01).

**Table 3:** Descriptive statistics of perioperative Outcome and complications on the overall population and after stratification for the type of PC-RPLND.

	**Overall (*n* = 171)**	**Template (*n* = 90)**	**Bilateral (*n* = 81)**	***p*-value**
Median operative time, min (IQR)	210 (150–240)	180 (150–210)	240 (180–329)	<0.001
Median intraoperative blood loss, ml (IQR)	500 (200–1500)	300 (100–700)	1400 (400–2450)	<0.001
Median length of hospital stay, days (IQR)	9 (8–10)	8 (7–10)	9 (8–12)	0.01
Adjunctive surgery, n (%)	56 (33%)	21 (23%)	35 (43%)	0.006
**CLAVIEN-DINDO COMPLICATION**				
0	94 (55%)	62 (69%)	32 (39%)	<0.001
I	19 (11%)	11 (12%)	8 (10%)	0.6
II	48 (28%)	14 (16%)	34 (42%)	<0.001
IIIa	5 (3%)	2 (2%)	3 (4%)	0.9
IIIb	0 (0%)	0 (0%)	0 (0%)	–
IVa	4 (2%)	1 (1%)	3 (4%)	0.5
IVb	0 (0%)	0 (0%)	0 (0%)	–
V	1 (1%)	0 (0%)	1 (1%)	0.9

Overall, 63 adjunctive procedures were necessary in 56 patients (33%). With 43% compared to 23%, adjunctive surgery was significantly more often performed in the bilateral resection group (*p* = 0.006). All kinds of adjunctive surgery were seen more frequently in bilateral resection group but only nephrectomy being significantly more often (*p* = 0.03, Table 4).

**Table 4:** Descriptive statistics of the type of adjunctive surgery on the overall population and after stratification for the type of PC-RPLND.

	**Total (*n* = 171)**	**Template (*n* = 90)**	**Bilateral (*n* = 81)**	***p*-value**
Aortic replacement	4 (2.3%)	0 (0%)	4 (5%)	0.1
Cava prothesis	3 (1.8%)	1 (1%)	2 (2.5%)	0.9
Cavotomy	14 (8.2%)	9 (10%)	5 (6%)	0.4
Bone resection	10 (5.8%)	5 (6%)	5 (6%)	0.9
Liver resection	19 (11.1%)	7 (8%)	11 (14%)	0.2
Nephrectomy	13 (7.6%)	3 (3%)	10 (12%)	0.03

The rate of high-grade postoperative complications (Clavien-Dindo ≥ III) was 6% in both groups. One Clavien-Dindo V complication (pulmonary embolism) was recorded 2 days after PC-RPLND. Four patients had to be transferred to intensive care unit (ICU) due to acute renal failure and had to undergo dialysis (Clavien-Dindo IVa). Two patients suffered from fascial dehiscence and required surgical wound revision, three patients developed hydronephrosis requiring ureteral stent placement.

A nerve sparing approach was performed in 115 of 171 patients (67%). In patients undergoing unilateral template resection preservation of sympathetic nerves could be achieved in 78 of 90 patients (87%). In bilateral template resection, nerve-sparing was performed in 36 of 81 patients (44%).

An interim analysis on ejaculation preservation for patients undergoing unilateral template resection between 2008 and 2015 showed antegrade ejaculation in 80% of all patients. From the current database we can provide additional data on ejaculation preservation for 37/171 (22%) patients. Of these 37 patients, 19 and 18 patients underwent bilateral or unilateral template resection, respectively. An antegrade ejaculation was seen in 37% (7/19) of patients in the bilateral resection group compared to 94% (17/18) in the unilateral template resection group.

### Histopathological Findings

The histological report of patients undergoing template resection showed teratoma in 44 patients (49%), necrosis in 38 patients (42%) and vital tumor in 8 patients (9%). In patients undergoing bilateral resection, teratoma was found in 43 patients (53%), necrosis in 31 patients (38%), and vital tumor in 7 patients (9%). There was no significant difference in histopathological findings between unilateral template and bilateral resection group (Table 5).

**Table 5:** Descriptive statistics on the PC-RPLND histologic report on the overall population and after stratification for the type of resection.

	**Overall (*n* = 171)**	**Template (*n* = 90)**	**Bilateral (*n* = 81)**	***p*-value**
Necrosis	69 (40%)	38 (42%)	31 (38%)	0.6
Teratoma	87 (51%)	44 (49%)	43 (53%)	0.6
Vital tumor	15 (9%)	8 (9%)	7 (9%)	0.9

### Follow-Up

The mean and median follow-up was 23 and 14.5 months (IQR 3.3–37.6). For 31 patients we could not provide follow-up data longer than 90 days postoperatively. Moreover, 79 patients presented with a follow-up longer than 1 year and 58 patients with a follow-up longer than 2 years. Thirty and Twenty-eight patients with at least 2-year follow up underwent bilateral and template resection, respectively.

During follow-up, a total of 23 (16.4%) recurrences were reported. Eighteen patients (12.9%) were treated with bilateral resection and 5 patients (3.6%) with template resection. Three patients with bilateral resection developed an infield recurrence and one patient with template resection an outside field recurrence. Of these 23 patients experiencing a relapse of disease 13 were initially classified as “poor prognosis” according to IGCCCG. Eight patients were initially “intermediate prognosis” and two patients were “good prognosis” (Table 6).

**Table 6:** Recurrences after PC-RPLND.

**Patient**	**Template**	**Site of relapse**
1.	Bilateral	Lung
2.	Bilateral	Liver/marker
3.	Bilateral	Marker
4.	Bilateral	Marker
**5**.	**Bilateral**	**Retroperitoneal (infield)**
6.	Template	Lung
7.	Template	Medistinal
8.	Bilateral	Retroperitoneal (outfield)
**9**.	**Bilateral**	**Retroperitoneal (infield)**
10.	Template	Retroperitoneal (opposite side)
11.	Template	Lung/skull
12.	Bilateral	Marker
13.	Bilateral	Lung
14.	Bilateral	Lung
15.	Bilateral	Mediastinal/Retrocrural
16.	Bilateral	Neck
**17**.	**Bilateral**	**Retroperitoneal (infield)**
18.	Bilateral	Retrocrural/Mediastinal
19.	Bilateral	Lung
20	Bilateral	Retrocrural
21.	Bilateral	Lung
22.	Bilateral	Lung
23.	Template	Marker

**Figure 2:**
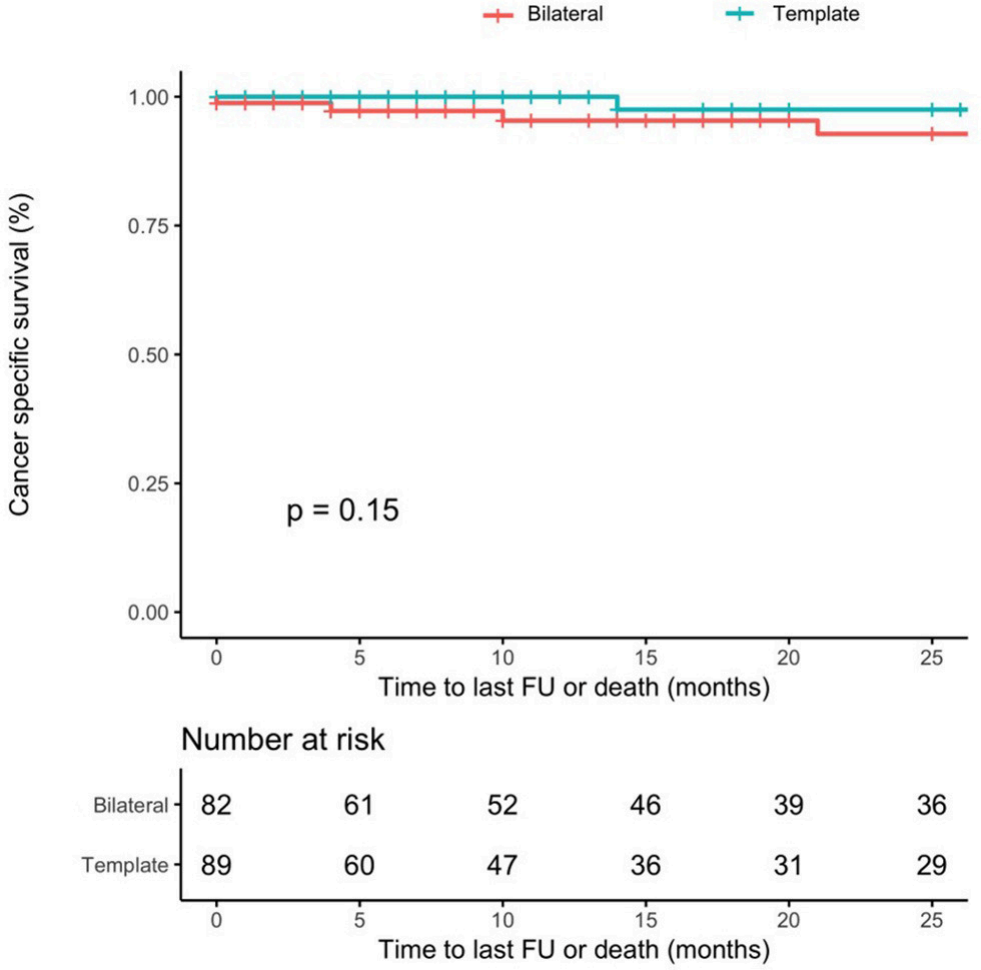
Cancer-specific mortality-free survival rates after PC-RPLND after stratification for type of resection (template–blue line vs. bilateral–red line, *p*-value = 0.15).

The 1-year and 2-year CSM-free survival rates were 100 and 97% for template, and 95 and 93% for bilateral resection, respectively (*p* = 0.15) (Figure 2).

**Figure 3:**
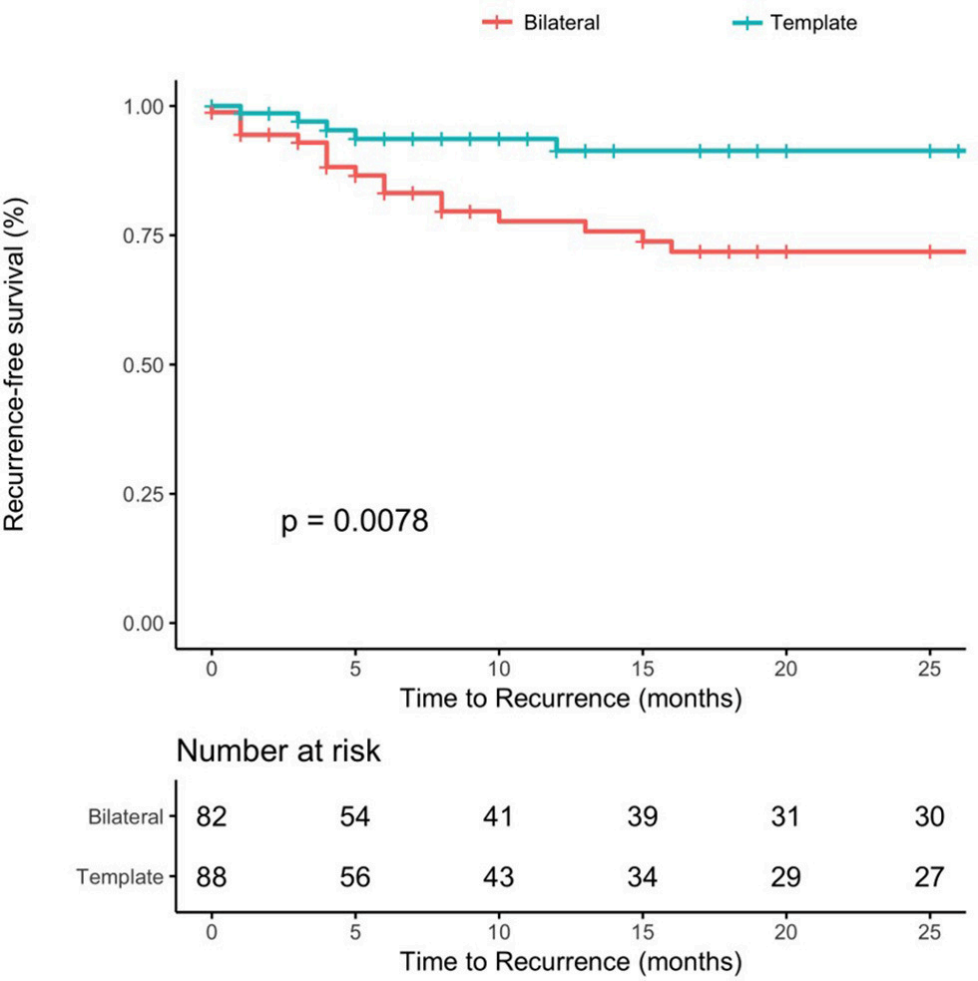
Recurrence-free survival rates after PC-RPLND after stratification for type of resection (template–blue line vs. bilateral–red line, *p*-value = 0.0078).

One-year and 2-year recurrence-free survival rates for patients with template resection were 91%, for patients undergoing bilateral resection 77 and 72%, (*p* = 0.0078) (Figure 3). The median time to recurrence was 9 and 9.5 months in the bilateral and template group, respectively.

Eight patients died due to progressive disease during follow-up. Seven of these eight patients underwent bilateral resection. Seven patients presented with clinical stadium III, one patient stadium II. Four and four patients were classified “intermediate” and “poor prognosis” at diagnosis. One patient died instantly after surgery due to lung embolism and one patient died of suicide.

## Discussion

Retroperitoneal lymph-node dissection plays a crucial role in treatment of non-seminomatous germ cell cancer and is mandatory in patients with a residual mass larger than 1 cm (22). Even in patients with a residual mass <1 cm, PC-RPLND seems to have a therapeutic role, since recurrent disease is seen in up to 9% of cases (22, 23).

In patients undergoing PC-RPLND, all areas of primary tumor should be completely resected. The current guidelines of the EAU and NCCN recommend to perform a nerve-sparing bilateral approach (7, 8) even though there is growing evidence that a modified template resection does not harbor a higher risk of relapse for selected patients. Perioperative risks and complications might as well be reduced by performing a modified unilateral approach (7, 14). Since more patients present with low-volume retroperitoneal disease at diagnosis, a full bilateral approach can be questioned (24). The purpose of the current study was to analyze perioperative and oncological outcomes of 171 patients after primary PC-RPLND for non-seminomatous germ cell cancer undergoing bilateral or unilateral template resection.

During a median follow-up of 14.5 months, 23 patients (16%) developed recurrences. In the bilateral resection group, we found three retroperitoneal recurrences, one of these in the pancreas. Two recurrences occurred after incomplete resection in a patient with bulky disease and inferior vena cava tumor thrombus. One patient with template surgery showed recurrent disease on the contralateral site. With a recurrence-free survival of 91% after 2 years for patients after unilateral template resection our data resembles those published by Heidenreich (1) and Beck et al. (13) who showed a 93.9 and 95% recurrence-free survival rate for unilateral template resections after 24 and 32 months, respectively. No recurrences were found within the resection field in the Beck study group and in one patient in the Heidenreich group (1, 13). Our data therefore support the concept that a bilateral PC-RPLND is not required in all patients.

In our analysis patients with a residual mass of <5 cm were considered for template resection. As one of the main reasons for unilateral resection is preserving antegrade ejaculation, we challenged the 5 cm rule in well selected cases and performed unilateral resection even if residual mass was >5 cm (e.g., in strictly unilateral tumor masses and when intraoperative findings allowed an oncological safe unilateral template resection). In the present cohort, only one retroperitoneal relapse after unilateral template resection was recorded. This patient showed a post-chemotherapy tumor size of 12 cm.

Aprikian and coworkers performed template PC-RPLND in 40 patients taking advantage of intraoperative frozen sections (14). In cases of necrosis or fibrosis, a template resection and in cases of teratoma bilateral resection were performed. Cho et al. (25) showed that, at 5 years, recurrence-free survival rates were 93% with no infield recurrences for patients with residual masses >5 cm and without teratoma in histopathological finding. All together these findings support the concept that template resection is oncological safe in terms of cancer-specific and recurrence-free survival for well selected patients (dimension <5 cm and absence of teratoma).

One major lack of the present study is the incomplete remission status for patients with stage III disease after PC-RPLND. As 69% of the cohort was classified as clinical stage III, not all patients underwent PC-RPLND for residual mass(es) in the chest, and even if surgery was performed data about oncological follow-up is frequently missing. Renunciation of PC-RPLND was either based on a standard CT scan without residual masses after chemotherapy or necrosis in final histopathology of retroperitoneal PC-RPLND. However, in this study we compare patients with complete remission status (CR) to patients with partial remission without elevated tumor markers (PRm-) which might be a bias. Furthermore, if we match our data to the literature, a higher mortality rate was expected. The current literature depicts a cancer-specific survival rate for “poor prognosis” patients up to 48%. In our patient cohort the cancer-specific survival rates of “poor prognosis” patients are higher (77%). This might be explained by the fact that all patients were treated at a tertiary high-volume center as well as the short follow-up of only 14.5 months and missing follow-up data for 31 patients.

The recommendation for a bilateral resection is based on the observation that in up to 8% microscopic metastasis are found in the contralateral template (19). One of the main objectives to perform template resection is to preserve antegrade ejaculation in these young patients to improve quality of life. In this series with limited follow-up, we did not find an increased recurrence rate, neither ipsilateral nor contralateral. The natural history of left-over teratoma remains unclear, so longer follow-up is necessary to support this observation.

Adjunctive surgery was performed in 56/171 patients (33%) and were seen significantly more often in bilateral PC-RPLND (43%) than in unilateral template resection (23%, *p* = 0.006). Heidenreich and colleagues presented in a series of 185 PC-RPLND patients an adjunctive surgery rate of 13.5% (26). In our series we saw a higher amount of adjunctive procedures with a rate of 33%. This might be explained both by the high percentage of patients classified as “poor prognosis” in our cohort (37 vs. 18.7%) and the correlation between adjunctive surgery rates and the IGCCCG classification and residual mass size (27). Nevertheless, a high-grade Clavien-Dindo complication rate of 6% in the present series reflects the published data by Heidenreich and Paffenholz (26, 28) and confirms that during bilateral PC-RPLND for a higher proportion of patients, adjunctive procedures are needed. However, when this kind of surgery is performed at a specialized tertiary referral center, high-grade Clavin-Dindo complication rates are low.

## Conclusion

Antegrade ejaculation in patients undergoing unilateral template resection with a residual mass <5 cm can be preserved at a much higher rate. Moreover, this surgical procedure is oncologically safe in terms of mid-term recurrence and CSM-free survival rates. This data therefore undermines the growing evidence of limited PC-RPLND being justifiable in strictly unilateral residual mass <5 cm. Due to worse preoperative risk factors, adjunctive procedures are more frequently performed during bilateral resection. This data has to be confirmed with a longer follow-up regarding in-field and retroperitoneal recurrences.

## Author Contributions

AH, AL, and PA contributed conception and design of the study. AH, AN, RgS, CW, and AF organized the database and the postal follow-up. AN performed the statistical analysis. AH wrote the first draft of the manuscript. All authors contributed to manuscript revision, read and approved the submitted version.

### Conflict of Interest Statement

The authors declare that the research was conducted in the absence of any commercial or financial relationships that could be construed as a potential conflict of interest. The handling editor declared a past co-authorship with one of the authors AN.
